# Responses of beneficial *Bacillus amyloliquefaciens* SQR9 to different soilborne fungal pathogens through the alteration of antifungal compounds production

**DOI:** 10.3389/fmicb.2014.00636

**Published:** 2014-11-21

**Authors:** Bing Li, Qing Li, Zhihui Xu, Nan Zhang, Qirong Shen, Ruifu Zhang

**Affiliations:** ^1^National Engineering Research Center for Organic-based Fertilizers, Jiangsu Collaborative Innovation Center for Solid Organic Waste Resource Utilization, Nanjing Agricultural UniversityNanjing, China; ^2^Key Laboratory of Microbial Resources Collection and Preservation, Ministry of Agriculture, Institute of Agricultural Resources and Regional Planning, Chinese Academy of Agricultural SciencesBeijing, China

**Keywords:** *Bacillus amyloliquefaciens* SQR9, transcriptional response, soilborne pathogens, lipopeptide antibiotics, bacteria-fungal interaction

## Abstract

*Bacillus amyloliquefaciens* SQR9 exhibited predominantly antagonistic activities against a broad range of soilborne pathogens. The fungi-induced SQR9 extracts possess stronger antifungal activities compared with SQR9 monoculture extracts. To investigate how SQR9 fine-tunes lipopeptides (LPs) and a siderophore bacillibactin production to control different fungal pathogens, LPs and bacillibactin production and transcription of the respective encoding genes in SQR9 were measured and compared with six different soilborne fungal pathogens. SQR9 altered its spectrum of antifungal compounds production responding to different fungal pathogen. Bacillomycin D was the major LP produced when SQR9 was confronted with *Fusarium oxysporum*. Fengycin contributed to the antagonistic activity against *Verticillium dahliae kleb*, *Fusarium oxysporum*, *Fusarium solani*, and *Phytophthora parasitica*. Surfactin participated in the antagonistic process against *Sclerotinia sclerotiorum*, *Rhizoctonia solani*, and *Fusarium solani*. Bacillibactin was up-regulated when SQR9 was confronted with all tested fungi. The reduction in antagonistic activities of three LP and bacillibactin deficient mutants of SQR9 when confronted with the six soilborne fungal pathogens provided further evidence of the contribution of LPs and bacillibactin in controlling fungal pathogens. These results provide a new understanding of specific cues in bacteria-fungi interactions and provide insights for agricultural applications.

## Introduction

*Bacillus* species are used as biocontrol agents for the suppression of many soilborne plant pathogens. *Bacillus* biocontrol activities involve a number of mechanisms, such as competition, antagonism, induction of systemic resistance, and promoting of plant growth. Many *Bacillus* strains potentially produce numerous antibiotics, which have begun to be screened. The direct use of these antibiotics is an attractive alternative control strategy to the use of living organisms. Indeed, the production of antibiotics plays a major role in disease suppression (Fravel, 1988; Dowling and O'Gara, 1994; Eshita et al., 1995). *B. amyloliquefaciens* FZB42 was reported to dedicate 8.5% of its genome for the synthesis of secondary metabolites (Chen et al., 2007), including production of lipopeptides (LPs), which were reported to be versatile weapons for plant disease biocontrol (Ongena and Jacques, 2008).

Interestingly, the expressions of the antibiotics synthesis genes appear to be regulated during microbe-microbe interactions in the environment. Under carbon-limited condition, 14.5% of the genes had higher expression in mixed cultures than in monocultures by random arbitrary primed-PCR (RAP-PCR) and suppressive subtractive hybridization (SSH). These genes were involved in secondary metabolites and multidrug resistance (Garbeva and de Boer, 2009). Antibiotic compound production could be triggered when antagonistic soil bacteria were confronted with other microorganisms (Becker et al., 1997). Garbeva et al. (2011) reported the regulation of various antibiotics in *Pseudomonas fluorescens* Pf0-1 when confronted with different bacterial competitors. Barret et al. (2009) showed that the association of the pathogenic fungus *Gaeumannomyces graminis* var. *tritici* with wheat roots strongly altered biocontrol bacterium *P. fluorescens* Pf29Arp adaptation, with differences between early- and late-infection stages. DeCoste et al. (2010) evaluated the effect of pathogenic *Verticillium dahliae* on the expression of *hcnC*, responsible for the production of hydrogen cyanide (HCN), which is important for the biological control of plant pathogens of *Pseudomonas* sp. LBUM300 in rhizosphere soil. Quantitative PCR results showed that the presence of *V. dahliae* had a significant stimulatory effect on *hcnC* expression and also increased the population of *Pseudomonas* sp. LBUM300. Jousset et al. (2011) focused on the interactions among plants and soil microorganisms and found that infection with the pathogen *Pythium ultimum* increased the expression of the antifungal gene *phlA* in the biocontrol strain *P. fluorescens* CHA0, indicating that communication with rhizosphere bacteria is involved in the pathogen response of the bacterial strain (Rosado et al., 1994; Raaijmakers et al., 1999; Mootz et al., 2001).

In the rhizosphere, the ability of plant growth-promoting rhizobacteria to inhibit the growth of a range of competing microbial species is essential for them to grow and survival (Whipps, 2001; Nihorimbere et al., 2011). Plant growth-promoting rhizobacterial strains, including many species of *Bacillus*, play an important role in the ecological control of soilborne pathogens. *Bacillus* produces LPs, which are versatile inhibitors of soilborne fungal pathogens in the rhizosphere (Ongena and Jacques, 2008). The iturin family encompasses the closely related cyclic LPs, iturins and the bacillomycins that have strong antifungal and hemolytic activities but only limited antibacterial activities (Thimon et al., 1992). As an antifungal peptide (Moyne et al., 2001), bacillomycin D has been shown to exert strong antifungal activity (Chen et al., 2007). Fengycin was reported as a novel antifungal LP produced by *Bacillus subtilis* F-29-3 (Vanittanakom et al., 1986), with antibiotic activity against filamentous fungi but not against yeast or bacteria. Surfactin had antimicrobial properties *in vitro* and could be involved in biocontrol functions in the rhizosphere (Thimon et al., 1992; Bais et al., 2004). Bacillibactin is produced by non-ribosomal synthesis and mediates iron transport in *Bacillus subtilis* (Dertz et al., 2006).

*B. amyloliquefaciens* SQR9, isolated from the rhizosphere of cucumbers, is a plant growth-promoting rhizobacteria with outstanding antimicrobial activity. SQR9 has been applied as a biocontrol agent for the suppression of soilborne plant pathogenic organisms (Cao et al., 2011). The antagonistic function of SQR9 has been attributed to the production of several secondary metabolites with antimicrobial activities (Xu et al., 2013, 2014). Bacillomycin D was identified in SQR9 as major compound to fight against *Fusarium oxysporum* f.sp. *cucumerinum*, and contribute to the SQR9 biofilm formation (Xu et al., 2013). LPs are recognized for the antifungal activities, but their antifungal spectrums and how are they regulated by specific fungal pathogen are not well known.

In this study, the LPs and a siderophore bacillibactin productions and the transcription expression of the respective encoding genes in SQR9 were measured when the strain was confronted with six different soilborne fungal pathogens. The results indicated that their productions and gene expressions were affected by pathogen, suggesting that SQR9 can distinguish between different competitors and fine-tune its strategy by using different antibiotics when faced with different fungal pathogens.

## Materials and methods

### Strains and growing conditions

*B. amyloliquefaciens* strain SQR9 (CGMCC accession no. 5808, China General Microbiology Culture Collection Center) was used throughout this study. *Bacillus* strains were cultivated on GB media (Glucose, 10 g; Difco peptone, 10 g; Merck meat extract, 2 g; Difco yeast extract, 1 g; NaCl, 5 g; Difco agar, 15 g; distilled water, 1 L; pH 7.0) solidified with 1.5% agar (Rosado et al., 1994). All bacterial strains were pre-cultured from frozen glycerol stocks on one-tenth strength Tryptic Soy Broth agar. The tested fungi *Verticillium dahliae Kleb* (CFCC accession no. 82516, China Forest Culture Collection Center), *Sclerotinia sclerotiorum* (Lib) De Bary (CFCC accession no. 83749, China Forest Culture Collection Center), *Fusarium oxysporum* CIPP1012 (ACCC accession no. 30220, Agricultural Culture Collection of China), *Rhizoctonia solani* (ACCC accession no. 36246, Agricultural Culture Collection of China), *Fusarium solani* (Mart.) App. *etwoll*. (CFCC accession no. 84587, China Forest Culture Collection Center), and *Phytophthora parasitica* var. *nicotianae* (Bredade Hean) *Tucker* (ACCC accession no. 36286, Agricultural Culture Collection of China) were grown on potato dextrose agar (PDA) (Table 1). Agar disks (1 cm in diameter) containing hyphae from the growing zone of mycelia, cultured on half-strength PDA, were placed inverted on PDA plates. The plates were sealed with parafilm and incubated for 7 days at 28°C.

**Table 1:** **Microorganisms and plasmids used in this study**.

**Strains**	**Characteristics**	**References or sources**
**BACTERIA**		
*E.coli* DH5α	*supE44, Δ lacU169 (φ 80lacZΔ M15), hsdR17, recA1, endA1, gyrA96, thi-1, relA1*	Invitrogen (Shanghai)
*B. amyloliquefaciens* SQR9	Wild type, soil isolate, gram-positive	Cao et al., 2011
*B. amyloliquefaciens* SQR9M1	*B. amyloliquefaciens* SQR9bmyD::Tc^r^	Xu et al., 2013
*B. amyloliquefaciens* SQR9M2	*B. amyloliquefaciens* SQR9fenA::Tc^r^	Xu et al., 2013
*B. amyloliquefaciens* SQR9M4	*B. amyloliquefaciens* SQR9srfAA::Crm^r^	This study
*B. amyloliquefaciens* SQR9M5	*B. amyloliquefaciens* SQR9dhbA::Crm^r^	This study
*B. amyloliquefaciens* SQR9M6	*B. amyloliquefaciens* SQR9sfp::Erm^r^	This study
**FUNGI**		
*Verticillium dahliae Kleb*	Pathogens of *Verticillium wilt*	Griffiths, 1971
*Sclerotinia sclerotiorum* (Lib.) De Bary	The causal agent of stem rot of oilseed rape	Behnam et al., 2006
*Fusarium oxysporum* f. sp. *Cucumerinum*	The causal agent of stem rot of *Fusarium* wilt of cucumber	Cao et al., 2011
*Rhizoctonia solani*	Cucumber *Rhizoctonia* rot	ACCC no. 36246
*Fusarium solani (Mart.)App.etwoll*.	Pepper root rot	CFCC no.84587
*Phytophthora parasitica* var. *nicotianae*	*P. nicotianae* var. *nicotianae* Waterhouse	ACCC no. 36286
**PLASMIDS**		
pAX01	Erm^r^	From BGSC
PBR322	Crm^r^	From BGSC

*^a^Amp^r^, Ampicillin resistance; Crm^r^, Chloramphenicol resistance; Erm^r^, Erythromycin resistance*.

### DNA transformation

SQR9 was electric-transformed using the method of Alexandre Rosado et al. (1994) with modifications. SQR9 cells were pre-cultured in LB medium, a fresh colony was inoculated into 3 mL LB liquid medium. After incubation in 37°C, 170 rpm overnight, 1 mL culture was transferred into a 500 mL Erlenmeyer flask containing 100 mL LB liquid medium. Then the flask was incubated at 37°C under the living condition until the OD_600_ = 0.5, then SQR9 cells were harvest, washed twice with PEB solution, and healed with 1 mL PEB solution. Subsequently, 100 ng DNA fragment were added to 0.2 ml of cell suspension and electroporation of cells was conducted. Then 0.8 mL LB liquid medium was added to recovery the transformants after incubation at 37°C under the living condition for 7 h. Finally, the cells were cultivated overnight in LB medium with the concentrations of the appropriate antibiotic.

### Genome analysis of NRPS genes in SQR9

Based on the whole-genome of SQR9 (NCBI accession No. CP006890), BLASTP was used to obtain function of all the protein-coding genes of SQR9 against reference genomes of *B. amyloliquefaciens* FZB42 (Chen et al., 2007) and *B. subtilis* 168 (Kunst et al., 1997) (parameters: *e*-value: 1e-5, coverage >60%, identity >50%). Homologous proteins, which were related to the LPs, and siderophore synthesis proteins and their genes (gene clusters) were identified in SQR9.

### Confrontation assays of SQR9 against fungi *in vitro*

The interaction experiments were performed using the modified PDA medium with 15 g of glucose instead of 20 g per l. An agar plug of growing fungi (0.5 cm in diameter) was placed in the center of a modified PDA plate and incubated for 1 day. SQR9 was then inoculated onto each of three equidistant points 2 cm from the plug. Three replicates were conducted. After 4 days of incubation, pictures were taken.

### Extraction of antifungal compounds *in vitro*

The extraction of antifungal compound(s) from agar was performed as described by Raaijmakers et al. (1999) and conducted in modified PDA medium. Briefly, the bacterial lawn growing on the agar plate was washed off by sterile ddH_2_O and OD_600_ was determined. The whole agar media of the plate was collected for next extraction. Sixteen plates were pooled as a replicate, and three batches (biological replicates) of 16 plates were extracted independently. The agar media were cut into small (approximately 1-cm diameter) pieces for a better extract efficiency. The agar pieces were vigorously shaken in 80% (v v^−1^) acetone for 1 h at room temperature; the volume of acetone added was determined by OD_600_ of former washed cells, reaching 1 × 10^9^ cells mL^−1^. Then, 200 mL of each sample solution were taken for further extract processing. The acetone solution was centrifuged for 10 min at 4000 × g, and the acetone was evaporated under airflow. The water fraction was acidified with trifluoroacetic acid [0.1% (v v^−1^)], mixed with two volumes of ethylacetate and shaken vigorously for 5 min. After incubation overnight at −20°C, the unfrozen (ethylacetate) fraction that contained the active compounds was carefully transferred to a new flask and dried under airflow. The dried extracts were dissolved in 150 μL of 50% (v v^−1^) methanol and subjected to reverse phase high pressure liquid chromatography analysis (see Antifungal assay of extractions).

### Antifungal assay of extractions

The extract solutions were tested for activities against growth of *Verticillium dahliae Kleb*., *Sclerotinia sclerotiorum*, *Fusarium oxysporum* f. sp., cucumerinum, *Rhizoctonia solani*, *Fusarium solani* (Mart.) App. etwoll., and *Phytophthora parasitica* in 12-well open-chamber plates (Cat No. 665180 Greiner bio-one, Frickenhausen, Germany) with 500 μL of modified PDA agar in each well. Then Each well was inoculated on one side with a 6 mm diameter agar disk containing fungal hyphae. A volume of 10 μL of the extract was added to sterile filter paper (Whatman No.1, Whatman Nederland BV, Hertogenbosch, The Netherlands; 6 mm diameter) and placed on the opposite side of the wells. Three replicates were conducted. The 12-well plates were incubated at 28°C for 48 h and checked for fungal growth inhibition by measuring distance from the mycelium to the filter paper. Filter paper disks containing 10 μL of 100% methanol, SQR9 and fungi monoculture agar extractions were used as controls.

### Phase high pressure liquid chromatography (HPLC) analyses of extractions

HPLC analysis was performed using a HPLC 1200 device (1200 series, Agilent, USA) (Raza et al., 2009). For the detection of LPs, a 20 μL sample pretreated using an XAD-16 column was injected into the HPLC column (Eclipse XDB-C18, 4.6 × 250 mm, 5 μm, Agilent). The purification was performed using a solvent containing A [0.1% (v v^−1^) CH_3_COOH] and B (CH_3_CN) at a flow rate of 0.6 mL min^−1^. An ultraviolet (UV) detector was used to detect peaks at 210 nm. The elution conditions were as follows: from 0 to 9 min, 60 to 93% (v v^−1^) mobile phase A plus 40–7% (v v^−1^) mobile phase B; from 9 to 20 min, 93% solvent B and 7% solvent A. The temperature was maintained at 35°C. Three replicates were detected out of each biological replicates. After HPLC detection, the peak area of each tested active compounds was analyzed.

### RNA isolation and cDNA synthesis

Total RNA was extracted from SQR9 cells growing on PDA plates with or without (control) the presence of fungi. The SQR9 colonies were harvested from the plates and dissolved in suitable volumes of sterile phosphate buffer solution (PBS) to obtain the same optical density (OD_600_) for each RNA extraction. RNA was extracted using the AxyPrep Multisource Total RNA Miniprep Kit (Axygen Biosciences 33210 Central Avenue, Union City, USA) according to the manufacturer's recommendations. The RNA concentrations were quantified using a NanoDrop Spectrophotometer (Nanodrop 2000, Thermo scientific, USA). The extracted total RNA was treated with the PrimeScript RT reagent Kit With gDNA Eraser (Perfect Real Time) (TaKaRa, Dalian, China). RNA was reverse transcribed into cDNA in a 20 μL reaction volume using a reverse transcription system (TaKaRa, Otsu, Shiga, Japan) according to the manufacturer's instructions. Three replicates were extracted for statistical analysis.

### Quantification of LPs and siderophore synthesis genes by qPCR

Quantitative PCR (qPCR) was performed to quantify gene transcription. Conventional PCR was also done with the RNA extracts to test for DNA contamination. A volume of 2 μL of cDNA was subjected to qPCR using SYBR Green PCR master mix (Applied Biosystem, Warrington, UK). For each target gene, primer sets (Table S1) were designed using Primer Premier 5 software (PREMIER Biosoft). All primers used for qPCR were first tested using conventional PCR with DNA isolated from *B. amyloliquefaciens* SQR9. qPCR was performed using an ABI 7500 system (ABI, USA) under the following conditions: initial cycle at 95°C for 10 s and 40 cycles at 95°C for 5 s, 65°C for 34 s, and 60°C for 34 s. The 2^−∆∆Ct^ method was used to analyze the qPCR data, and *recA* was used as reference gene (Livak and Schmittgen, 2001). Three replicates were conducted out of three replicated templates for further statistic analysis.

### Construction and antagonistic testing of SQR9 mutants

Based on the qPCR analyses, two LPs and a siderophore encoding genes and the *sfp* gene, which was necessary all LPs synthesis in *B. amyloliquefaciens* SQR9 were deleted. The SQR9M1 and SQR9M2 mutant of *B. amyloliquefaciens* SQR9 deficient of bacillomycin D and fengycin production, respectively, were constructed and included in this study (Xu et al., 2013).

To disrupt surfactin synthesis in SQR9, a 1049 bp fragment upstream of *srfAA* was PCR obtained using primer P19 and P20, (Table S1, all the primers mentioned later can be found in Table S1) and the 1163 bp *srfAA* downstream fragment was amplified with primer set P23/P24, using SQR9 genome as template, which was extracted using Axygen Multisource Genomic DNA Miniprep Kit (Axygen Scientific, Inc., USA). The chloramphenicol resistance cassette was amplified by P21/P22, using plasmid PBR322 as the template; 35 bp of primer P21 and P22 were overlapped with upstream and downstream fragments of *srfAA* gene, respectively. Overlap PCR was used to splice the *srfAA* gene upstream fragment, chloramphenicol resistance cassette and the downstream fragment with primer set P25/P26. The fused PCR fragment was transformed into *B. amyloliquefaciens* SQR9 for homologous recombination. The disruption of *srfAA* was verified in the resistant colonies using primer sets P19/P22 and P21/P24 and the correct mutant was designated as SQR9M4.

The bacillibactin encoding gene *dhbA* and the *sfp* gene were disrupted by using the same strategy. For *dhbA* gene, primer sets P27/P28 and P31/P32 were used to get the upstream and downstream fragments, respectively. Primer set P29/P30 was used to amplify the chloramphenicol resistance cassette, and primer set P27/P32 was used to fuse the three fragments. The disruption of *dhbA* was verified in the resistant colonies using primer sets P27/P30 and P29/P32 and the correct mutant was designated as SQR9M5.

For disruption of the *sfp* gene, the upstream and downstream fragments were amplified using primer sets P11/P12 and P15/P16, respectively. The erythromycin resistant cassette was got from plasmid pAX01 with primer set P13/P14. The three fragments were fused by an overlap PCR using primer set P11/P16. The disruption of *sfp* was verified in the resistant colonies using perimer sets P11/P14 and P13/P16 and the correct mutant was designated as SQR9M6.

The SQR9 mutants were compared with the wild-type strain for LP and bacillibactin production using Landy medium. Confrontation assays of SQR9 wild type and mutants toward soilborne pathogens were conducted using extracted antagonistic substances. For the production of antagonistic substances, *B. amyloliquefaciens* SQR9 and mutants were grown in 500-mL Erlenmeyer flasks with 100 mL of Landy broth (Landy et al., 1948) incubated at 30°C under the living condition for 60 h. The culture was centrifuged at 13,000 g (4°C) for 20 min, and the supernatant were collected. To prepare the samples for further antagonistic activity experiments, 50 mL of the supernatant was passed through an Amberlite XAD-16 (Alfa Aesar, a Johnson Matthey Company, WardHill, MA) column (10 g). A gradient of 100% methanol was used to remove the bound solute. Elution was dried in a rotation evaporator (<40°C) and dissolved in 15 mL methanol. The solution was then filtered through a 0.22 μm hydrophilic membrane to give the final antimicrobial extract solution (Yuan et al., 2012).

The extractions of SQR9 mutants were tested for their antifungal abilities using PDA plates as described above. An agar plug of growing fungal strain (0.5 cm in diameter) was placed in the center of the plate and incubated for 1 day. The antimicrobial extracts (50 μL) were placed into wells on the agar at three equidistant points 2 cm from the plug. After 4 days of incubation, pictures were taken. Methanol was used as a negative control.

### Statistical analysis

For each experiment, three replicates were included. The statistical analyses of qPCR data were carried out with SPSS software (SPSS Inc., Chicago, IL, USA) and subject to One-Way ANOVA analysis, means were analyzed by the Duncan's multiple range tests at *P* = 0.05.

## Results

### *B. amyloliquefaciens* SQR9 produced multiple lipopeptide compounds suppressing a broad range of fungal pathogens

**Figure 1:**
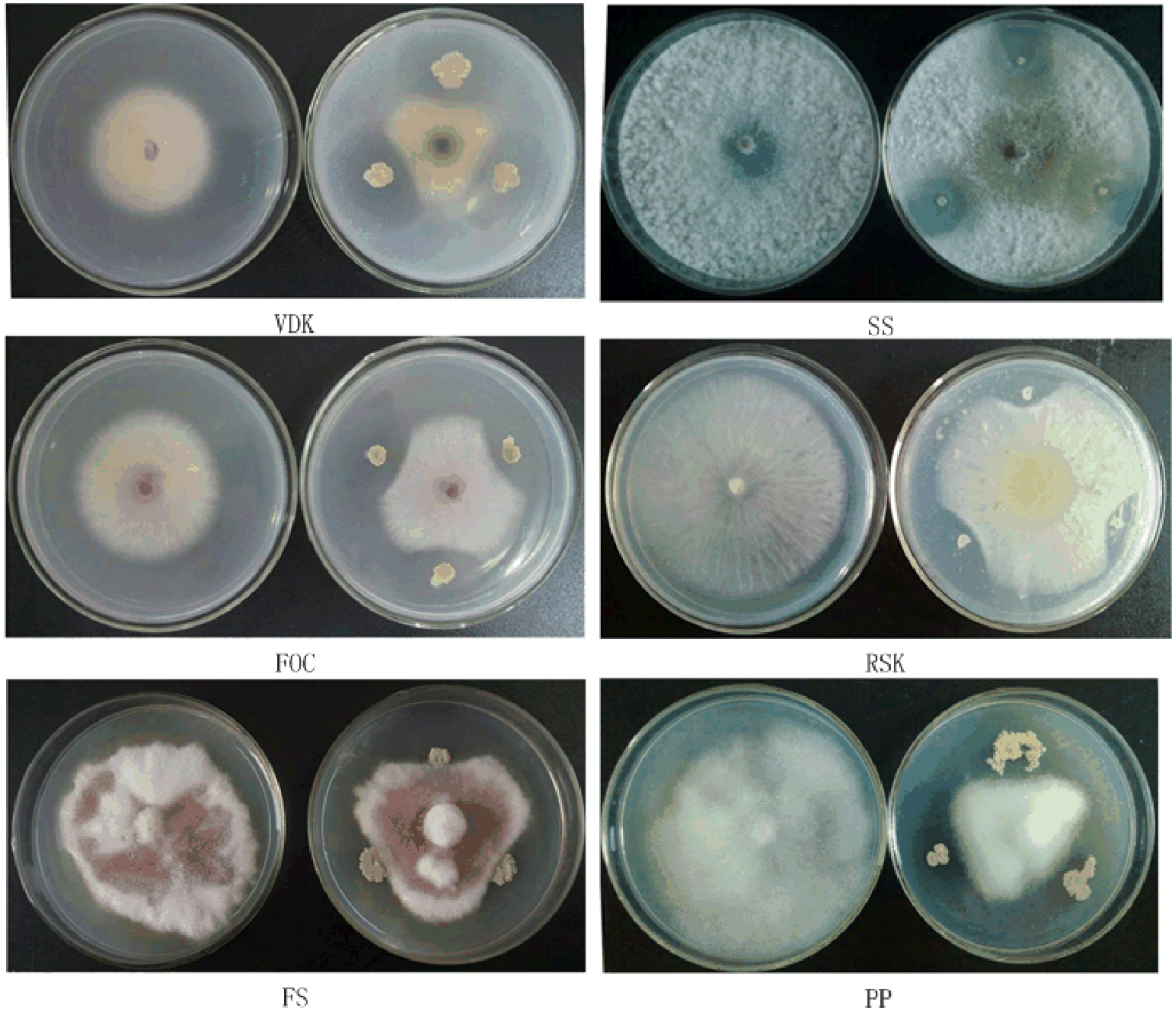
**Antimicrobial spectrum of SQR9 with 6 tested soil-borne pathogens**. VDK, *Verticillium dahliae Kleb*; SS, *Sclerotinia sclerotiovum* (Lib.) *de Bary*; FOC, *Fusarium oxysporum* f. sp. *cucumerinum*; RSK, *Rhizoctonia solani Kahn*.; FS, *Fusarium solani* (Mart.)App.*etwoll*.; and PP, *Phytophthora parasitica* var.*nicotianae* (Bredade Hean) *Tucker*.

**Figure 2:**
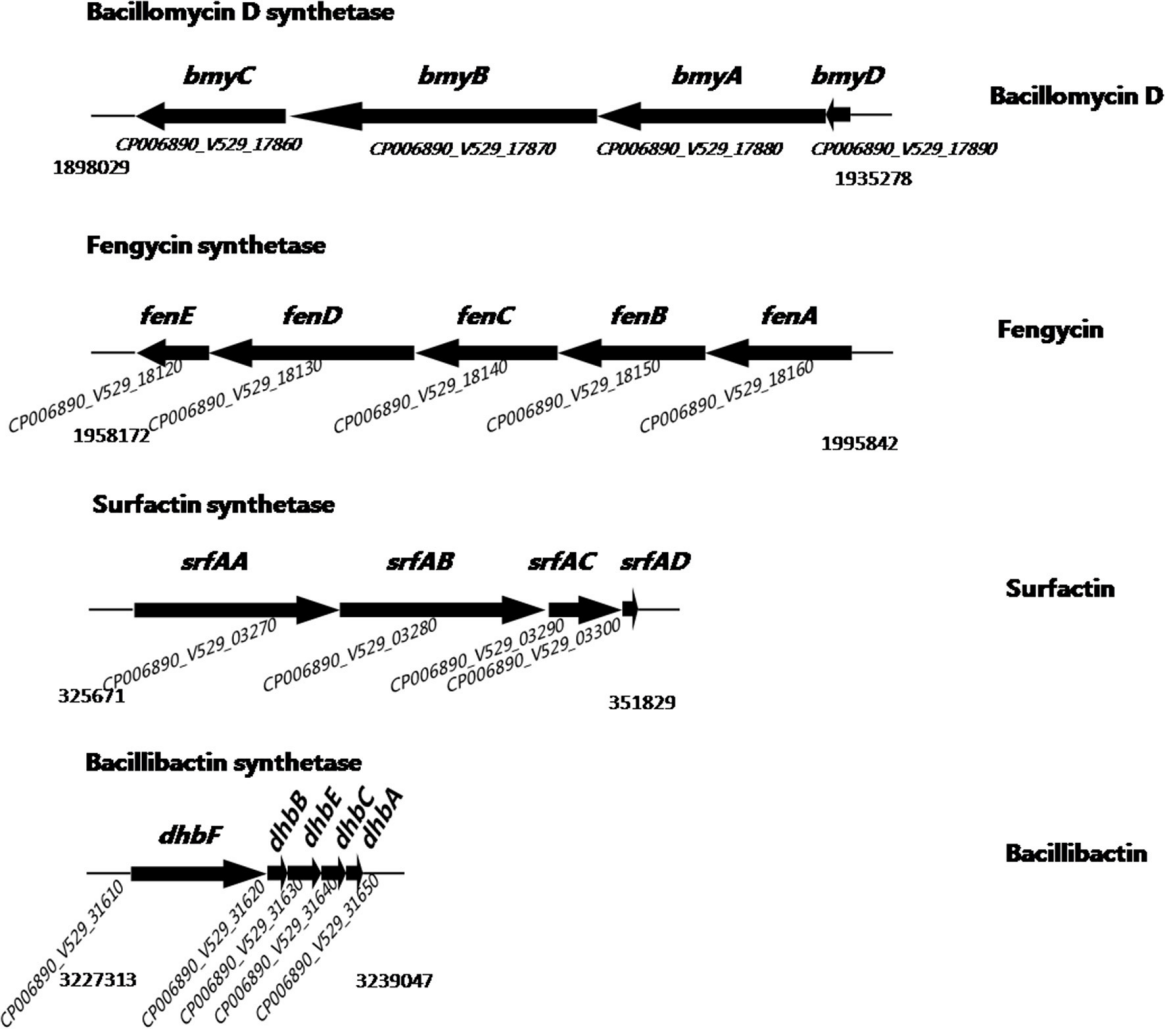
**Schematic representation of secondary metabolite gene clusters in *Bacillus amyloliquefaciens* SQR9**. Gene clusters encoding non-ribosomal peptide synthetases (NRPSs). The names assigned to individual genes in SQR9 are indicated above the arrows. Products assigned to the respective pathways are shown on the right. GenBank accession numbers in the SQR9 genome are indicated below the arrows.

The SQR9 colonies developed inhibition zones against the fungal pathogens on PDA agar (Figure 1), indicating that SQR9 was effective against a broad range of fungal pathogens. Our previous research detected the LPs and the siderophore bacillibactin in the supernatant of the SQR9 (Xu et al., 2013, 2014). Genomic analysis of SQR9 identified their gene clusters (*bmyDABC*, *fenE-A*, *srfAA-AD*, and *dhbE-A*) responsible for the synthesis of bacillomycin D, fengycin, surfactin and bacillibactin, respectively (Figure 2, Table S2).

**Figure 3:**
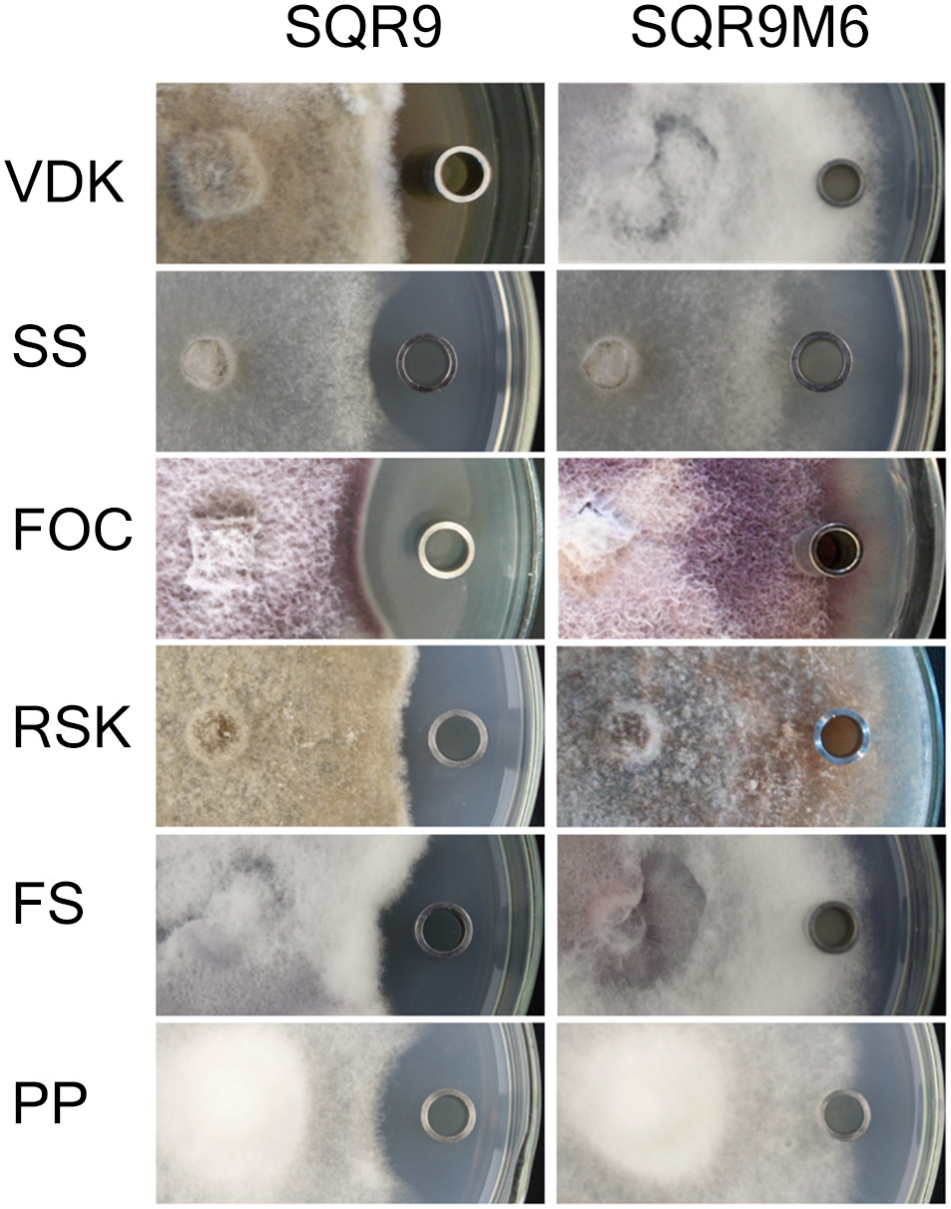
**Antagonistic assay of extractions of the Sfp-deficient mutant SQR9M6 against 6 fungal pathogens**. **Left**, oxford cup containing extraction from SQR9 monoculture. **Right**, Oxford cup containing extraction from Sfp-deficient mutant SQR9M6. VDK, *Verticillium dahliae Kleb*; SS, *Sclerotinia sclerotiorum*; FOC, *Fusarium oxysporum*; RSK, *Rhizoctonia solani* (Mart.) App.*etwoll*; FS, *Fusarium solani*; and PP, *Phytophthora parasitica*.

To verify that LPs and bacillibactin were involved in the antifungal activities, *sfp* gene deficient mutant SQRM6 were tested against six fungal pathogens since the synthesis of both LPs and the siderophore is reported to depend on the presence of a functional *sfp* gene coding for a 4-phosphopantetheinyl transferase (Mootz et al., 2001; Chen et al., 2007). Results showed strain SQR9M6 lost antifungal activities against all tested fungi (Figure 3). HPLC analysis showed that bacillomycin D, fengycin, surfactin and bacillibactin were not detected in strain SQR9M6 (Table S3), confirming that the LPs and bacillibactin of SQR9 were responsible for the suppression of fungal pathogens.

### *B. amyloliquefaciens* SQR9 antagonistic activity was induced by interactions with the pathogen

Extracts collected in the presence of fungi showed significant difference in the antagonistic activity compared with extracts from the SQR9 monoculture. Negative controls (extraction of fungi monoculture and methanol) showed no antagonistic activity against fungal pathogens. The strongest antagonistic activity against a particular pathogen was from extracts produced in the presence of that fungus (Table 2). These results revealed that the bacterium produces a different spectrum of antifungal compounds in response to different fungal species.

**Table 2:** **Antagonist assay against fungal pathogens**.

**Fungi**	**Distance of fungi plug to filtered paper (mm)**						
	***m***	***a***	***b***	***c***	***d***	***e***	***f***
VDK	1.87 ± 0.09^b^	6.34 ± 0.11^e^	2.36 ± 0.04^d^	1.52 ± 0.19^a^	2.29 ± 0.07^c^	1.52 ± 0.08^a^	2.63 ± 0.05^d^
SS	0.81 ± 0.06^a^	1.23 ± 0.09^b^	3.62 ± 0.11^f^	2.21 ± 0.12^d^	1.53 ± 0.14^c^	1.71 ± 0.05^c^	3.29 ± 0.06^e^
FOC	2.54 ± 0.11^e^	2.51 ± 0.06^e^	1.46 ± 0.05^b^	3.32 ± 0.16^f^	1.91 ± 0.09^c^	2.35 ± 0.06^d^	0.84 ± 0.11^a^
RSK	1.17 ± 0.06^a^	3.56 ± 0.07^cd^	3.47 ± 0.11^c^	3.15 ± 0.09^b^	7.51 ± 0.03^e^	3.69 ± 0.14^d^	3.25 ± 0.02^b^
FS	0.52 ± 0.03^a^	0.87 ± 0.03^b^	1.81 ± 0.04^e^	1.44 ± 0.05^d^	1.06 ± 0.02^c^	2.41 ± 0.03^f^	1.66 ± 0.05^e^
PP	0.49 ± 0.05^b^	1.31 ± 0.13^d^	0.83 ± 0.05^c^	0.30 ± 0.05^a^	0.54 ± 0.12^b^	0.34 ± 0.04^a^	1.93 ± 0.06^e^

*Distance of fungi plug to filter paper is in millimeters. Extraction of SQR9 monoculture is represented as m, extractions of SQR9 agar confronted with VDK, Verticillium dahliae Kleb; SS, Sclerotinia sclerotiorum; FOC, Fusarium oxysporum; RSK, Rhizoctonia solani; FS, Fusarium solani; PP, Phytophthora parasitica var. nicotianae are represented as a, b, c, d, e, and f, respectively. Data represent the mean value ± standard error from three replicates. The data obtained were subjected to One-Way ANOVA analysis and means were analyzed by the Duncan's multiple range tests at P ≤ 0.05*.

### *B. amyloliquefaciens* SQR9 antifungal compounds production and transcription of their encoding genes were enhanced by interaction with the pathogen

HPLC analysis of bacillomycin D, fengycin, surfactin and bacillibactin in the extracts of the SQR9 monoculture and the fungal pathogen confronted SQR9 showed different patterns of LPs and bacillibactin production (Table 3). Increases in bacillomycin D, fengycin and bacillibactin production were detected when SQR9 was confronted with *Verticillium dahliae Kleb*. Bacillomycin D, surfactin and bacillibactin were up-regulated when SQR9 was confronted with *Sclerotinia sclerotiorum*. Compared with SQR9 monoculture, the production of bacillomycin D, fengycin and bacillibactin was higher when SQR9 was faced with *Fusarium oxysporum*. When SQR9 was confronted with *Rhizoctoniasolani* and *Fusarium solani*, the production of surfactin and bacillibactin increased. All the four compounds were increased when SQR9 was exposed to *Phytophthora parasitica*.

**Table 3:** **HPLC analysis of SQR9 when confronted with different fungal pathogens**.

**LPs**	**Peak areas of 4 antibiotics (mAU/s)**						
	**M**	**VDK**	**SS**	**FOC**	**RSK**	**FS**	**PP**
Bacillomycin D	26542 ± 286^c^	52637 ± 290^e^	55143 ± 247^f^	51741 ± 584^e^	22550 ± 1056^b^	2021 ± 172^a^	30889 ± 180^d^
Fengycin	4666 ± 58^d^	12709 ± 71^g^	4015 ± 20^c^	5084 ± 18^e^	3624 ± 71^b^	2677 ± 62^a^	7753 ± 47^f^
Surfactin	3617 ± 77^c^	3302 ± 104^b^	9040 ± 84^g^	2846 ± 45^a^	7640 ± 110^f^	5622 ± 74^e^	4294 ± 78^d^
Bacillibactin	6933 ± 19^a^	10523 ± 24^g^	8267 ± 41^d^	7621 ± 18^b^	8911 ± 8^e^	9717 ± 10^f^	7796 ± 84^c^

a−b *Means and standard deviations were calculated from peak areas of SQR9 classified into different antibiotic production. Small letters in brackets indicate significant differences as determined by One-Way ANOVA and Duncan's multiple range tests*.

*Peak areas of 4 antibiotics, mAU/s as unit. Extraction of SQR9 monoculture is represented as m, extractions of SQR9 agar confronted with VDK, Verticillium dahliae Kleb; SS, Sclerotinia sclerotiorum; FOC, Fusarium oxysporum; RSK, Rhizoctonia solani; FS, Fusarium solani; PP, Phytophthora parasitica var. nicotianae, respectively. Data represent the mean value ± standard error from three replicates. The data obtained were subjected to One-Way ANOVA analysis and means were analyzed by the Duncan's multiple range tests at P ≤ 0.05*.

Transcriptional profiles of the four antifungal compounds in SQR9 were evaluated by qPCR for further investigation. Gene transcription (Table 4) showed similar patterns (Table 3); *fenA* (in the fengycin operon) was up-regulated when SQR9 was confronted with *Verticillium dahliae Kleb* and *Fusarium oxysporum*; *bmyD* (in the bacillomycin D operon) was up-regulated by *Sclerotinia sclerotiorum* and *Fusarium oxysporum*; *srfAA* (in the surfactin operon) was up-regulated when SQR9 was confronted with *Sclerotinia sclerotiorum*, *Rhizoctonia solani*, and *Fusarium solani*; *dhbA* (in the bacillibactin operon) was up-regulated through SQR9 interaction with *Verticillium dahliae Kleb*, *Fusarium oxysporum*, and *Rhizoctonia solani*. All four genes were up-regulated by *Phytophthora parasitica*.

**Table 4:** **Transcriptional data of SQR9 when confronted with different fungal pathogens**.

**Gene**	**Relative gene expression (fold change)**						
	**M**	**VDK**	**SS**	**FOC**	**RSK**	**FS**	**PP**
*bmyD*	0.247 ± 0.006^c^	0.243 ± 0.004^c^	1.732 ± 0.002^d^	2.603 ± 0.006^f^	0.180 ± 0.001^b^	0.120 ± 0.006^a^	1.990 ± 0.001^e^
*fenA*	0.924 ± 0.006^c^	12.807 ± 0.006^f^	0.920 ± 0.001^c^	2.932 ± 0.002^d^	0.80 ± 0.001^b^	0.640 ± 0.001^a^	3.210 ± 0.001^e^
*srfA*	0.860 ± 0.001^b^	0.672 ± 0.003^b^	3.763 ± 0.005^e^	0.111 ± 0.001^a^	3.053 ± 0.003^d^	1.488 ± 0.577^c^	1.551 ± 0.001^c^
*dhbA*	0.885 ± 0.006^b^	15.121 ± 0.001^g^	2.692 ± 0.002^c^	10.233 ± 0.003^f^	3.295 ± 0.005^d^	0.88 ± 0.001^a^	4.732 ± 0.001^e^

a−b *Means and standard deviations were calculated from relative gene expression of SQR9 classified into different genes. Small letters in brackets indicate significant differences as determined by One-Way ANOVA and Duncan's multiple range tests*.

*QRT-PCR result of SQR9 monoculture is represented as m, QRT-PCR results of SQR9 confronted with Verticillium dahliae Kleb, Sclerotinia sclerotiorum, Fusarium oxysporum, Rhizoctonia solani, Fusarium solani, Phytophthora parasitica var. nicotianae are represented as VDK, SS, FOC, RSK, FS, and PP, respectively. B. amyloliquefaciens SQR9-recA was used as an internal reference gene. Data are expressed as log10 relative quantification (RQ) (−2^ΔΔ CT^). Data represent the mean value ± standard error from three replicates. The data obtained were analyzed by One-Way ANOVA analysis and means were analyzed by the Duncan's multiple range tests at P ≤ 0.05*.

### *B. amyloliquefaciens* SQR9 antifungal compounds production in deficient mutants revealed the major active compound toward a specific fungal pathogen

Bacillomycin D, fengycin, surfactin, and bacillibactin production by SQR9 mutants (SQR9M1, SQR9M2, SQR9M4, and SQR9M5, respectively) was tested for their antagonistic activities against the six fungal pathogens (Table 5, Figure S1). Each mutant strain produces all other antibiotics at wild type levels except that encoded by the lacking gene (Table S3).

**Table 5:** **Distance between the fungal mycelium and wall of oxford cup of SQR9 wild type or mutants**.

**Fungi**	**Distance between the mycelium and wall of oxford cup (cm)**				
	**9**	**M1**	**M2**	**M4**	**M5**
VDK	0.63 ± 0.05^d^	0.35 ± 0.03^c^	0.13 ± 0.03^b^	0.42 ± 0.05^c^	−0.39 ± 0.04^a^
SS	0.56 ± 0.03^d^	0.32 ± 0.03^c^	0.54 ± 0.04^d^	−0.81 ± 0.04^a^	0.07 ± 0.03^b^
FOC	0.83 ± 0.03^d^	−0.87 ± 0.04^a^	0.31 ± 0.03^b^	0.81 ± 0.02^d^	0.60 ± 0.06^c^
RSK	0.39 ± 0.04^d^	0.29 ± 0.02^c^	0.32 ± 0.03^cd^	−0.60 ± 0.04^a^	−0.01 ± 0.04^b^
FS	0.55 ± 0.06^c^	0.24 ± 0.07^b^	0.14 ± 0.04^b^	−0.29 ± 0.05^a^	−0.37 ± 0.05^a^
PP	0.77 ± 0.05^b^	0.29 ± 0.03^a^	0.25 ± 0.04^a^	0.31 ± 0.03^a^	0.29 ± 0.04^a^

a−b *Means and standard deviations were calculated from distances of each mutant classified into confrontation with different fungal pathogens. Small letters in brackets indicate significant differences as determined by One-Way ANOVA and Duncan's multiple range tests*.

*Antagonistic assay of extractions from SQR9 wild-type and mutant strains against 6 fungi. Data stands for distance between the mycelium and well. VDK, Verticillium dahliae Kleb; SS, Sclerotinia sclerotiorum; FOC, Fusarium oxysporum; RSK, Rhizoctonia solani; FS, Fusarium solani; and PP, Phytophthora parasitica; 9, extraction of SQR9 wild type; M1, extraction of SQR9M1, bacillomycin D deficient mutant; M2, extraction of SQR9M2, fengycin deficient mutant; M4, extraction of SQR9M4, surfactin deficient mutant; and M5, extraction of SQR9M5, bacillibactin deficient mutant. Three replicates were used. Data represent the mean value ± standard error from three replicates. The data obtained were subjected to One-Way ANOVA analysis and means were analyzed by the Duncan's multiple range tests at P ≤ 0.05.Negative value represented that the mycelium growing over the wall of oxford cup and how far it was away from the left wall (of the oxford cup)*.

When confronted with *Verticillium dahliae Kleb*, SQR9M2 decreased the antagonistic activity significantly, while SQR9M1 and SQR9M4 only decreased the activities slightly. The bacillibactin-deficient mutant SQR9M5 did not show inhibitory activity against *Verticillium dahliae Kleb*. (Table 5, Figure S1). These results indicated that bacillibactin is the most effective against *Verticillium dahliae Kleb*, while fengycin can also contribute to the suppression. SQR9M4 lost antagonistic activity against *Sclerotinia sclerotiorum*, while SQR9M1 and SQR9M5 only slightly decreased the activities, indicating that surfactin is essential in the suppression of *Sclerotinia sclerotiorum* (Table 5, Figure S1). Bacillomycin D contributes the most to the antagonism of SQR9 against *Fusarium oxysporum* because SQR9M1 almost lost antagonistic activity (Table 5, Figure S1). For pathogen *Rhizoctonia solani*, SQR9M4 lost all antifungal activity, and SQR9M5 showed weak activity compared to the wild-type SQR9 (Table 5, Figure S1), indicating that surfactin and bacillibactin were important in the antagonistic process of SQR9 against *Rhizoctonia solani*. SQR9M4 and SQR9M5 lost antibiotic activity against *Fusarium solani* indicating surfactin and bacillibactin were responsible for suppression of *Fusarium solani*. (Table 5, Figure S1). When tested against *Phytophthora parasitica*, all four mutants showed reduced antagonistic activities compared with the SQR9 wild-type strain (Table 5, Figure S1), which meant that all four compounds were involved in the biological control of against *Phytophthora parasitica*.

## Discussion

SQR9 had a broad spectrum of antifungal ability *in vitro*. Extractions from cultures treated with *Verticillium dahliae Kleb* had stronger antibiotic activity against *Verticillium dahliae Kleb* compared with the other five fungal-induced extracts. The same pattern existed among the other five pathogens and their induced extracts, indicating that the antifungal compounds production in SQR9 is responsive to the specific fungal species.

When faced with six fungal pathogens, SQR9 antifungal compounds production and the transcription of their encoding genes shared the similar pattern. The up-regulated genes participated in the antagonistic activity of SQR9 toward the corresponding fungal strain, the activity differed when SQR9 were confronted with different fungal pathogens. These results indicated that interactions between SQR9 and the fungal pathogen have evolved to give an efficient antibiotic production to an efficient survival in the environment. SQR9 had different antifungal compounds production profiles, suggesting that the inhibition of pathogens by SQR9 is a fine-tuned process. Combination therapy is clearly an accepted part of clinical practice in the antibiotic use (Silver and Bostian, 1993). In this study, *B. amyloliquefaciens* SQR9 used multiple antifungal compounds to suppress a specific fungal pathogen. These combinations depended on confrontation with the fungal pathogen and had a species-specific response. Evidence for interspecies communication between these particular pathogens is especially intriguing, owing to their origins and ecological attributes.

We studied the response of *B. amyloliquefaciens* SQR9 to different competitors in one-to-one confrontations. However, bacteria are likely to encounter several different competitors at the same time in natural settings (Hibbing et al., 2010). In such situations, the production of a broad-spectrum antibiotic would be a beneficial strategy for SQR9 to address diverse competitors. Although these assays are somewhat artificial, they do allow for an in-depth analysis of fundamental mechanisms underlying microbial interactions. Our results demonstrated that *B. amyloliquefaciens* SQR9 has a species-specific transcriptional and metabolic response to competitors, which may provide new insights in the identification of specific cues in bacteria-fungi interactions and of novel competitive strategies, antimicrobial traits and genes.

### Conflict of interest statement

The authors declare that the research was conducted in the absence of any commercial or financial relationships that could be construed as a potential conflict of interest.

## Abbreviations

LP (LPs): Lipopeptide (lipopeptides)
NRPS: Non-ribosomal peptide synthetase
HPLC: Phase high pressure liquid chromatography.
