# Supplementary material for: Responses of beneficial Bacillus amyloliquefaciens SQR9 to different soilborne fungal pathogens through the alteration of antifungal compounds production
Source: Front Microbiol. 2014 Nov 21;5:636. doi: 10.3389/fmicb.2014.00636 (PMC4240174; doi:10.3389/fmicb.2014.00636)

***Supplementary Material***

**Responses of beneficial *Bacillus amyloliquefaciens* SQR9 to different soilborne fungal pathogens through the alteration of antifungal compounds production**

**Bing Li^1^, Qing Li^1^, Zhihui Xu^1^, Nan Zhang^1^, Qirong Shen^1^, Ruifu Zhang^1,2^***

^1^National Engineering Research Center for Organic-based Fertilizers, and Jiangsu Collaborative Innovation Center for Solid Organic Waste Resource Utilization, Nanjing Agricultural University, Nanjing, 210095, P.R. China

^2^Key Laboratory of Microbial Resources Collection and Preservation, Ministry of Agriculture, Institute of Agricultural Resources and Regional Planning, Chinese Academy of Agricultural Sciences, Beijing 100081, P.R. China

*** Correspondence:**Ruifu Zhang,College of Resources & Environmental Science, Nanjing Agricultural University, 6 Tongwei Road, Nanjing, Jiangsu Province 210095, P.R China.

[rfzhang@njau.edu.cn](mailto:rfzhang@njau.edu.cn)

1. **Supplementary Figures and Tables**

## 1.1 Suplementary Tables

**Table S1** Primers used in this study

| **Primers** | **Sequence** | **Genes** |
| --- | --- | --- |
| P1 | 5-AGTCTAAGTATTGGCGAAACGA-3 | *bmyD* |
| P2 | 5-ATTATGCTGAAAGTGAAGGGCG-3 |  |
| P3 | 5-TGGATGGTTCCTCCGCTATCTA-3 | *fenA* |
| P4 | 5-GGTGACGACCGCGCATTTTATT-3 |  |
| P5 | 5-GCTTCGTTCACTTCACGGTAGG-3 | *sftA* |
| P6 | 5-ATGGAGGAAAGACTCGGCTTTT-3 |  |
| P7 | 5-TCGGATTTCTTTTGCCACTTGG-3 | *dhb* |
| P8 | 5-ATAACGGGCGCTCCTTCGGTTC-3 |  |
| P9 | 5-AAAAAACAAAGTCGCTCCTCCG-3 | *recA* |
| P10 | 5-CGATATCCAGTTCAGTTCCAAG-3 |  |
| P11 | 5-GATTGTCGTGCTCGTAGA-3 | *sfp*-up |
| P12 | 5-CTGCCGCTTGATTCATTC-3 |  |
| P13 | 5-CCTTTGAGGCGGACGCTGAATGAATCAAGCGGCAG TCTAGAGCAACGTTCTTGCCATTG-3 | *sfp*-Mid |
| P14 | 5-TCCGCACCGCTGCGGCGAAGGCTTACGGACTTGAT ACTCTTCCTTTTTCAATATTATTG-3 |  |
| P15 | 5-ATCAAGTCCGTAAGCCTTC-3 | *sfp*-down |
| P16 | 5-GCGGTTATGCTACAATGAC-3 |  |
| P17 | 5-CCATCTTCAGCAAACTCGCC-3 | *sfp*-fusion step2 primer |
| P18 | 5-CTTTCTGTCGGCCAATGGTC-3 |  |
| P19 | 5-ATGCTGATTATGTGGCACTTGGG-3 | *srfAA*-up |
| P20 | 5-CGCCGGACGAATTCCTGTATC-3 |  |
| P21 | 5-TACGTTGAGAAAGCGATACAGGAATTCGTCCGGCG  GCATAAAGTGTAAAGCCTGGGG-3 | *srfAA*-mid |
| P22 | 5-CATTCGGCTTCTCTGTGTCAGGGCTCAGCGATTGT AATGTGGAATTGGGAACGGAAA-3 |  |
| P23 | 5-ACAATCGCTGAGCCCTGACAC-3 | *srfAA*-down |
| P24 | 5-CCATCGTTTCTATGCCGTTGA-3 |  |
| P25 | 5-AAGTTTACCCTGTCGTCCCTC-3 | *srfAA*-fusion step2 primer |
| P26 | 5-GGTAGTCTTTCTTCTCTGATGCC-3 |  |
| P27 | 5-AAGCTCTCCCTTTACCTCGGTG-3 | *dhb*-up |
| P28 | 5-ATGATTTATGCGTTGATGGCGG-3 |  |
| P29 | 5-GCCTAAAGTAGCGCCGCCATCAACGCATAAATCAT GCATAAAGTGTAAAGCCTGGGG-3 | *dhb*-Mid |
| P30 | 5-AATATGGACGCATTGGGAATGAAAGGGAAAACGGC AATGTGGAATTGGGAACGGAAA-3 |  |
| P31 | 5-GCCGTTTTCCCTTTCATTCC-3 | *dhb*-down |
| P32 | 5-CGTATCTTTGTCTCTTTTCCTCA-3 |  |
| P33 | 5-GGCACATCAAGCACACGGCAGAA-3 | *dhb*-fusion step2 primer |
| P34 | 5-TTCCCCGTCATATACCTGCTCGA-3 |  |

**Table S2** Lipopeptides and siderophore gene clusters indentified in SQR9 genome

| SQR9 GID | reference GID | query  length | subject  length | identity | evalue | product |
| --- | --- | --- | --- | --- | --- | --- |
| V529_17860 | 154686249 | 2619 | 2619 | 0.98396 | 0 | BmyC |
| V529_17870 | 154686250 | 5363 | 5363 | 0.98023 | 0 | BmyB |
| V529_17880 | 154686251 | 3982 | 3982 | 0.98292 | 0 | BmyA |
| V529_17890 | 154686252 | 400 | 400 | 0.98 | 0 | BmyD |
| V529_18120 | 154686275 | 1267 | 1267 | 0.97474 | 0 | FenE |
| V529_18130 | 154686276 | 3591 | 3591 | 0.98246 | 0 | FenD |
| V529_18140 | 154686277 | 2549 | 2549 | 0.98156 | 0 | FenC |
| V529_18150 | 154686278 | 2565 | 2565 | 0.97973 | 0 | FenB |
| V529_18160 | 154686279 | 2552 | 2552 | 0.97061 | 0 | FenA |
| V529_03270 | 154684834 | 3584 | 3584 | 0.98689 | 0 | SrfAA |
| V529_03280 | 154684835 | 3586 | 3586 | 0.98912 | 0 | SrfAB |
| V529_03290 | 154684837 | 1278 | 1278 | 0.982 | 0 | SrfAC |
| V529_03300 | 154684838 | 243 | 243 | 0.98765 | 0 | SrfAD |
| V529_31610 | 154687302 | 2375 | 2375 | 0.98232 | 0 | DhbF |
| V529_31620 | 154687303 | 308 | 308 | 0.98377 | 0 | DhbB |
| V529_31630 | 154687304 | 541 | 541 | 0.99261 | 0 | DhbE |
| V529_31640 | 154687305 | 398 | 398 | 0.98241 | 0 | DhbC |
| V529_31650 | 154687306 | 261 | 256 | 0.9765625 | 7.33E-175 | DhbA |

**Table S3** HPLC analysis data of four mutants compared with SQR9 wild type

| LPs | Production of LPs valued by HPLC Peak Area (mAU/s) | | | | | |
| --- | --- | --- | --- | --- | --- | --- |
|  | SQR9 | M1 | M2 | M4 | M5 | M6 |
| Bacillomycin D | 276523.00±5465.43^a^ | N/D | 276622.67±13623.10^a^ | 282324.00±5135.22^a^ | 277559.00±5875.10^a^ | N/D |
| Fengycin | 6064.61±73.56^a^ | 6080.66±140.61^a^ | N/D | 6062.76±61.24^a^ | 6008.11±29.77^a^ | N/D |
| Surfactin | 1963.67±24.80^a^ | 1946.38±40.67^a^ | 1936.27±14.10^a^ | N/D | 1942.98±36.24^a^ | N/D |
| bacillibactin | 6979.01±34.60^a^ | 6919.34±115.05^a^ | 6979.70±51.23^a^ | 6983.14±65.94^a^ | N/D | N/D |

Note: Peak areas of 4 antibiotics of each mutant, mAU/s as unit. Extraction of SQR9 wild type was represented as SQR9 M1, M2, M4, M5, M6 represents the bacillomycin D deficient mutant SQR9M1, fengycin deficient mutant SQR9M2, surfactin deficient mutant SQR9M4 and bacillibactin deficient mutant SQR9M5, sfp deficient mutant SQR9M6, respectively.

Three replicates were used. Data represent the mean value ± standard error from three replicates. The data obtained were transformed and subject to one-way ANOVA analysis and means were analyzed by the Duncan’s multiple range tests at *P*≤0.05. N/D: not detected.

## Suplementary Figures

**Figure S1.** Antagonistic assay of extractions from SQR9 wild-type and mutant strains against 6 fungi. VDK: *Verticillium dahliae Kleb*, SC: *Sclerotinia sclerotiorum*, FOC: *Fusarium oxysporum*, RSK: *Rhizoctonia solani Kahn.*, FS: *Fusarium solani*, and PP: *Phytophthora parasitica*. SQR9. Extraction of SQR9 wild type, SQR9M1. extraction of SQR9M1, bacillomycin D deficient mutant; SQR9M2. extraction of SQR9M2, fengycin deficient mutant; SQR9M4. extraction of SQR9M4, surfactin deficient mutant; and SQR9M5. extraction of SQR9M5, bacillibactin deficient mutant.

**E**

***fen***

***bam***

***srfA***

***dhb***

**SQR9**


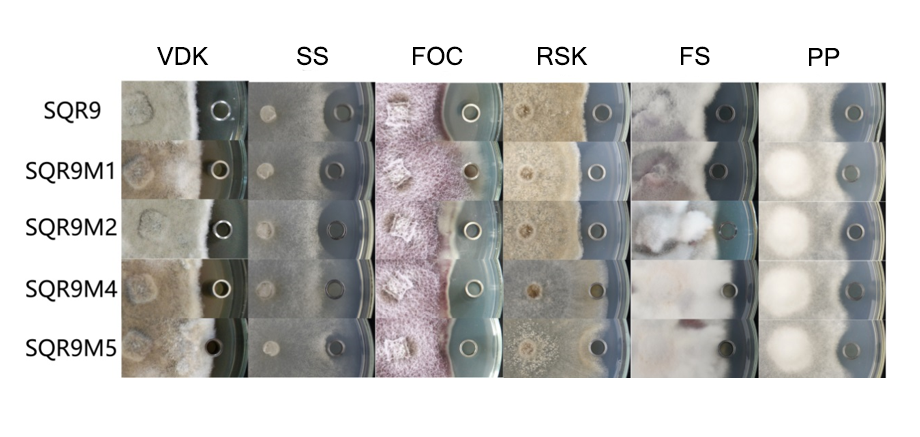


**Figure S2.** Reversed-phase HPLC chromatograms of lipopeptides produced by B. amyloliquefaciensSQR9 and sfp-deficient mutant SQR9M6. *B. amyloliquefaciens* SQR9 and its sfp-deficient mutant were grown in Landy medium at 30°C for 60 h, and the lipopeptides were extracted using XAD-16. The three NRPS peaks from left to right(A), and bacillibactin(B) are indicated.

A

B


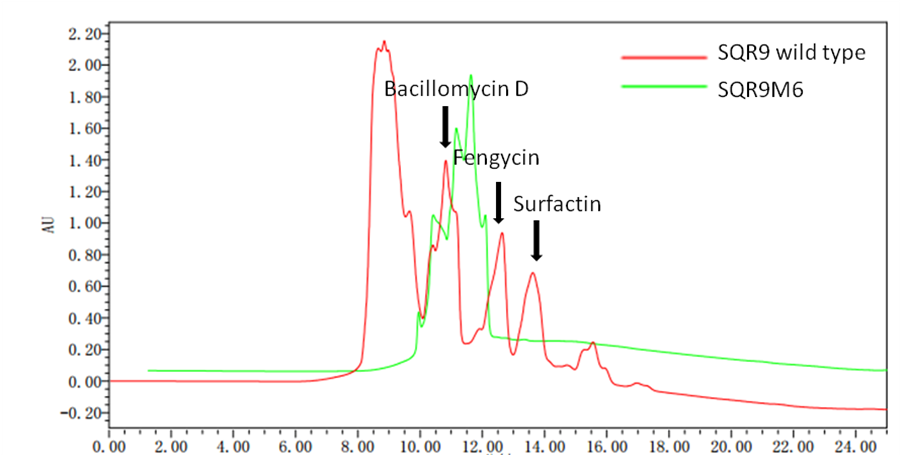

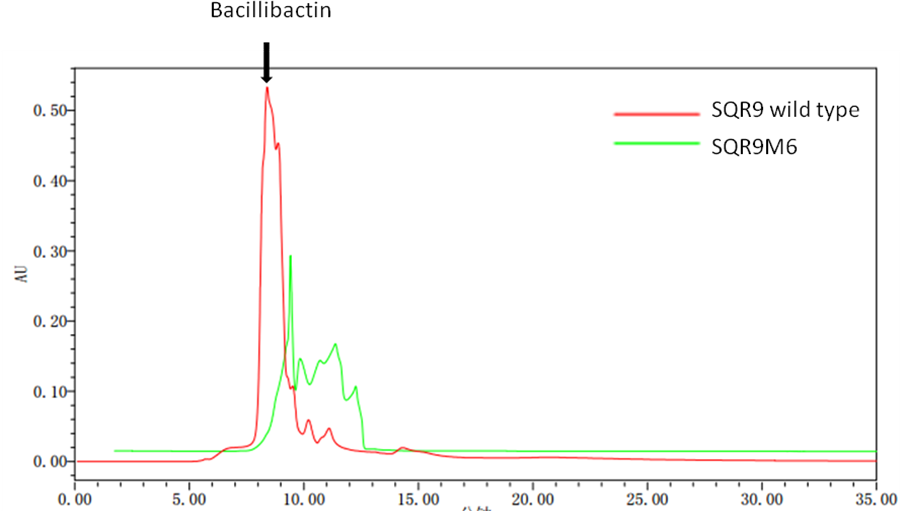

Supplement: Supplementary file 1 [file DataSheet1.DOCX]
